# Menstrual Blood as a Diagnostic Specimen for Human Papillomavirus Genotyping and Genital Tract Infection Using Next-Generation Sequencing as a Novel Diagnostic Tool

**DOI:** 10.3390/diagnostics14070686

**Published:** 2024-03-25

**Authors:** Hin-Fung Tsang, Yui-Shing Cheung, Chi-Shing Allen Yu, Chung-Sum Sammy Chan, Chi-Bun Thomas Wong, Kay-Yuen Aldrin Yim, Xiaomeng Pei, Sze-Chuen Cesar Wong

**Affiliations:** 1 Department of Clinical Laboratory and Pathology, Hong Kong Adventist Hospital, Hong Kong SAR, China; 2 Department of Obstetrics and Gynaecology, Queen Elizabeth Hospital, Hong Kong SAR, China; octoday@hotmail.com (Y.-S.C.); scschan@ha.org.hk (C.-S.S.C.); 3 Codex Genetics Limited, Hong Kong SAR, China; allenyu@codexgenetics.com (C.-S.A.Y.); thomas.wong@codexgenetics.com (C.-B.T.W.); aldrinyim@codexgenetics.com (K.-Y.A.Y.); 4 Department of Applied Biology & Chemical Technology, The Hong Kong Polytechnic University, Hong Kong SAR, China; xiaomeng.pei@connect.polyu.hk

**Keywords:** menstrual blood, metagenomic next-generation sequencing, 16S rRNA, genital tract infection, human papillomavirus, HPV, cervical cancer screening

## Abstract

Background: Menstrual blood (MB) is a convenient specimen type that can be self-collected easily and non-invasively by women. This study assessed the potential application of MB as a diagnostic specimen to detect genital tract infections (GTIs) and human papillomavirus (HPV) infections in women. Method: Genomic DNA was extracted from MB samples. Pacific Bioscience (Pacbio) 16S ribosomal DNA (rDNA) high-fidelity (HiFi) long-read sequencing and HPV PCR were performed. Results: MB samples were collected from women with a pathological diagnosis of CIN1, CIN2, CIN3 or HPV infection. The sensitivity and positive predictive value (PPV) of high-risk HPV detection using MB were found to be 66.7%. A shift in vaginal flora and a significant depletion in *Lactobacillus* spp. in the vaginal microbiota communities were observed in the MB samples using 16S rDNA sequencing. Conclusions: In this study, we demonstrated that MB is a proper diagnostic specimen of consideration for non-invasive detection of HPV DNA and genotyping using PCR and the diagnosis of GTIs using metagenomic next-generation sequencing (mNGS). MB testing is suitable for all women who menstruate and this study has opened up the possibility of the use of MB as a diagnostic specimen to maintain women’s health.

## 1. Introduction

Genital tract infections (GTIs) and human papillomavirus (HPV) infections are common in women of all age groups worldwide. Most bacterial GTIs and HPV infections in women are asymptomatic, and untreated GTIs could lead to adverse infection outcomes including pelvic inflammatory disease (PID), infertility, ectopic pregnancy, and preterm delivery, as well as congenital or neonatal infections [1]. New data are emerging that indicate women whose vaginal microbiota communities are largely depleted of *Lactobacillus* spp. are at higher risk of symptomatic bacterial vaginosis (BV), sexually transmitted diseases (STDs), and preterm birth. In contrast, women with vaginal microbiota communities dominated by *Lactobacillus* species are less likely to experience a number of adverse health outcomes [2]. An increased diversity of the vaginal microbiota, in combination with a reduced relative quantity of certain *Lactobacillus* species, was also found to be associated with a higher risk of HPV infections and the subsequent development of precancerous lesions and cervical cancer [2]. However, there are several reasons that discourage women from taking diagnostic tests of GTIs and HPV infections. For example, some women feel pain and discomfort during sample collection; some women feel embarrassed to remove their undergarments in front of another person; and some women do not feel comfortable letting anything be inserted into their genital tract due to some previous traumatic experiences and past sexual history [3].

The human endometrium undergoes more than 400 cycles of shedding over the whole reproductive period of a woman [4]. Menstruation is the monthly shedding of the lining of the uterus. As the lining of the uterus breaks down and sheds, menstrual blood (MB) containing blood and tissues from the uterus, as well as the infectious agents inside the reproductive system, flows from the uterus through the cervix and out of the body through the vagina. Based on the fact that MB carries the causative pathogens of GTIs and HPV when it passes through the genital tract during menstruation, we proposed to use MB as a non-invasive diagnostic specimen for HPV genotyping and GTI diagnosis. The advantages of using MB as a diagnostic specimen include (1) non-invasiveness of sample collection; (2) self-sampling is possible and (3) collection is simple and quick; and (4) easily performed by patients in private without any embarrassment during sample collection process [5]. Hence, the use of MB as a diagnostic specimen is suitable for all women who menstruate. Wong et al. have demonstrated the potential applications of MB as a diagnostic specimen for detecting HPV infection and premalignant cervical diseases (PCDs) non-invasively, in sexually active women [6,7].

**Figure 1:**
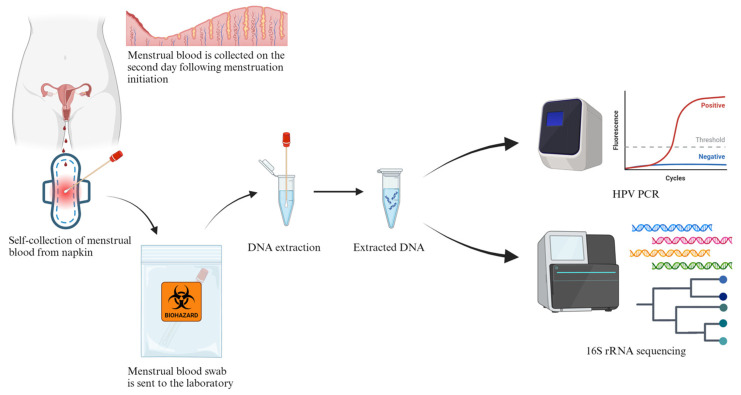
The workflow of menstrual blood collection and laboratory testing. This figure was created with BioRender (https://biorender.com) (accessed on 14 February 2024).

Metagenomic next-generation sequencing (mNGS) is a promising diagnostic approach that allows a hypothesis-free and comprehensive genetic analysis of the clinical specimens that cover nearly all pathogens [8,9]. In this study, in order to explore the potential application of MB as a diagnostic specimen to detect GTIs using mNGS, as well as HPV infections, we collected six MB specimens from sexually active women aged between 34 and 45 years. These collections were conducted at the Department of Obstetrics & Gynaecology, Queen Elizabeth Hospital, Hong Kong Special Administrative Region (HKSAR), China, between August and October 2022, to be tested by mNGS analysis using Pacific Bioscience (Pacbio) 16S ribosomal RNA coding gene (rDNA) high-fidelity (HiFi) sequencing. The long sequencing reads obtained from Pacbio HiFi sequencing allow high-quality and accurate species identification for mNGS analysis. In addition to 16S rDNA sequencing, HPV PCR was also performed on the MB samples to detect the presence of high-risk HPV. The demographic and clinical information of these patients are also reported. The workflow of MB collection and laboratory testing is shown in Figure 1.

## 2. Method and Materials

### 2.1. Patients

Six women aged between 34 and 45 years with a pathological diagnosis of cervical intraepithelial neoplasia (CIN) 1, CIN 2, CIN 3 or HPV infection were recruited at the Department of Obstetrics & Gynaecology, Queen Elizabeth Hospital, HKSAR, China, between August and October 2022. This study was approved by the Research Ethics Committee (Kowloon Central/Kowloon East), Hospital Authority, HKSAR (KC/KE-20-0200/ER-4). Written informed consent was obtained from all patients who participated in this study. The demographic and clinical information of the patients were obtained.

### 2.2. MB Collection and DNA Extraction

On the second day following menstruation initiation, participating patients were instructed to collect MB samples on used napkins with a collection foam swab (Puritan, Guilford, ME, USA). The MB swab was then sent to the laboratory and genomic DNA was extracted using a QIAamp^®^ DNA Blood Kit (Qiagen, Hilden, Germany) according to the manufacturer’s instructions. Extracted DNA was stored at −20 °C before analysis.

### 2.3. PacBio 16S rRNA HiFi Long-Read Sequencing

The purity and quantity of the extracted DNA was assessed using Nanodrop 2000 (Thermo Fisher Scientific, Waltham, MA, USA) and Qubit 4.0 (Thermo Fisher Scientific, Waltham, MA, USA). For library preparation, the PacBio 16S rRNA degenerate forward and reverse primer set, which is linked with a dual index barcode for demultiplex and sample identification, was used for construction of an indexed library for bacterial 16S rRNA gene (V1 to V9 regions) according to the PacBio standard protocol for the Amplification of bacterial full-length 16S gene with barcoded primers. A total of 14 indexed libraries were pooled together which underwent SMRTbell ligation by utilizing SMRTbell prep kit 3.0 (PacBio, Menlo Park, CA, USA). The SMRTbell ligated library pool was prepared for sequencing by using the Sequel^®^ II Binding Kit 3.1 (PacBio, Menlo Park, CA, USA) and in 1 SMRT cell for generation of high-fidelity long reads in the Sequel IIe system.

### 2.4. 16S rRNA Gene Analysis

The PacBio SMRT Portal was used to generate circular consensus sequences (CCS) from the raw reads, with a threshold of single-molecule basecalling accuracy of 99.9%. The HiFi reads were exported in FASTA format and quality filtering (Q20 or above) and trimming of sequencing adapters was performed using the cutadapt software (version 4.0). Despite the high accuracy of HiFi reads, amplicon errors which have accumulated during the library preparation process may still exist. Therefore, the QIIME2 (version 2022.2.1) [10] and DADA2 pipeline (version 1.22.0) [11] were used to generate high-quality denoised amplicon sequences and sequence variants in single-nucleotide resolution.

All processed reads were analysed using the QIIME2 and Mothur pipeline (version 1.39.5.0) [12]. First, trimmed reads were clustered into unique sequences. Next, the clustered sequences were aligned to the SILVA reference database (release 138) and any sequences that did not match the expected 16S amplicon coordinates were removed. Potential chimeric sequences were removed using the VSEARCH algorithm. After taxonomic classification using the RDP reference database (version 18) [13] and a Naïve-Bayes classifier, non-bacterial sequences were removed with a bootstrap confidence threshold of 80%. Finally, QIIME 2 was used for phylogenetic tree generation, summary results generation and plotting.

### 2.5. Menstrual Blood HPV PCR

HPV DNA detection and genotyping was performed on an ABI7500 real-time polymerase chain reaction genotyping platform using two commercial HPV DNA PCR assays, namely GeneProof Human Papillomavirus (HPV) Screen PCR Kit (GeneProof, Brno-jih, Czech Republic) and Yaneng Human Papillomavirus Nucleic Acid Detection and HPV16/18 Genotyping Kit (PCR-Fluorescence Probing) (Yaneng BIOscience, Shenzhen, China), according to the manufacturer’s instructions. The GeneProof kit can detect and identify high-risk HPV types 16, 18, 26, 30, 31, 33, 34, 35, 39, 45, 51, 52, 53, 56, 58, 59, 66, 67, 68, 69, 70, 73, 82, 97, whereas the Yaneng kit can detect and identify high-risk HPV types 16, 18, 26, 31, 33, 35, 39, 45, 51, 52, 53, 56, 58, 59, 66, 68, 73, 82.

## 3. Results

### 3.1. Patients’ Characteristics 

Six women aged between 33 and 45 years with pathological diagnosis of CIN 1, CIN 2, CIN 3 or HPV infection were recruited between August and October 2022. Three patients were diagnosed with low-grade squamous intraepithelial lesion (LSIL). Two patients were diagnosed with atypical squamous cells of undetermined significance (ASC-US). Two patients had atypical squamous cells, but high-grade squamous intraepithelial lesion could not be excluded (ASC-H). All patients in this study did not receive any HPV vaccination.

### 3.2. mNGS Analysis of MB

**Figure 2:**
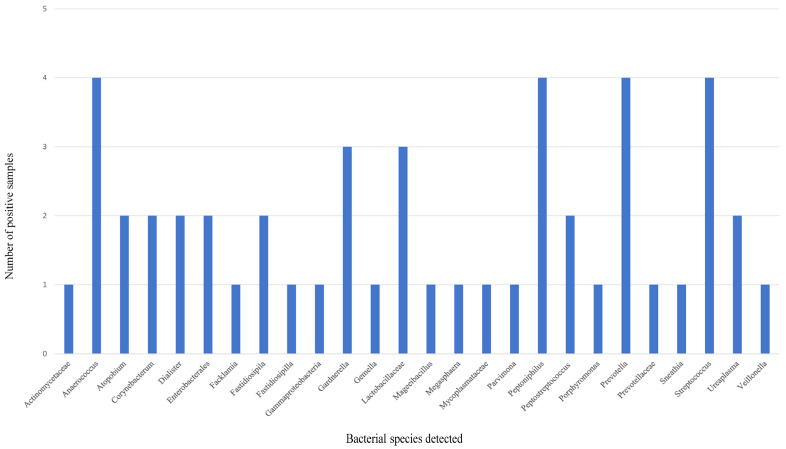
Bacterial species detected in the menstrual blood samples.

The summary statistics of PacBio HiFi sequencing data on MB samples are shown in Table 1. A shift in vaginal flora and a significant depletion in *Lactobacillus* species in the vaginal microbiota communities were observed in all participating patients through mNGS analysis of the MB samples (Table 2). An increase in microbiota diversity was also observed. According to community state types (CSTs) classification [14], all MB samples in this study belong to CST IV B. The most frequently detected bacteria in the MB samples from HPV-infected women were *Lactobacillaceae*, *Prevotella*, *Gardnerella*, *Peptoniphilus*, *Anaerococcus* and *Streptococcus* (Figure 2).

### 3.3. HPV DNA Detection of MB

High-risk HPV DNA was detected in four out of six MB samples (ST04, ST10, ST12 and ST13). Of the six patients who participated in this study, four of them were histologically confirmed to have HPV infection (ST06, ST10, ST12 and ST13). HPV DNA was detected in the MB samples of 80% (four out of five) patients who were diagnosed as CIN 1 to 3 histologically. On the other hand, HPV DNA was detected in the MB samples of 75% (three out of four) patients who were confirmed to be infected by HPV by HPV PCR in liquid-base papanicolaou (Pap) smear sample. In a patient (ST06) who was confirmed to have HPV infection histologically, high-risk HPV DNA was also detected in a Pap smear sample by PCR. However, HPV DNA was not detected in the MB sample from the same patient. In another patient (ST12), HPV infection was confirmed histologically. HPV DNA was not detected in the Pap smear sample by PCR, while HPV 16 DNA was detected in the MB sample (Table 2). Overall, the sensitivity and positive predictive value (PPV) of HPV detection using MB compared to HPV detection using Pap smear were found to be 66.7% in this study (Table 3).

## 4. Discussion

MB is a convenient specimen type that can be self-collected easily and non-invasively by women. In this study, we demonstrated the potential applications of MB as a diagnostic specimen for detecting HPV infection by PCR and GTIs by mNGS non-invasively in sexually active women. To the best of our knowledge, this is the first study using mNGS as a diagnostic tool with MB samples. Unlike previous studies which requested the patients to submit the whole used napkins in a zippered plastic bag to the laboratory for testing [5], the patients who participated in this study were instructed to collect MB on napkins with a collection foam swab only. The collection swab was then sent to the laboratory for genomic DNA extraction and further testing. This sample collection method provided patients with higher convenience and less hygiene problems during storage and transportation. It would fit better the real situation if MB testing were to be translated to routine clinical testing.

Cervical cancer is the fourth most common female cancer worldwide [15,16]. On the other hand, cervical cancer is a largely preventable disease that can be prevented by vaccination and screening. Studies have shown that early-stage detection of cervical cancer is associated with significantly improved survival rates. HPV is a non-enveloped double-stranded DNA virus that belongs to the *Papillomaviridae* family and *Firstpapillomavirinae* subfamily. There are 40 genotypes of HPV which can infect the human anogenital area. Among these genotypes, 14 of them are designated as high-risk HPV, which can cause several cancers in humans including cervical cancer. Low-risk HPV is related to mild squamous epithelial lesions and genitourinary warts. However, it is also found in cervical carcinoma sometimes [17]. HPV-encoded E6 and E7 proteins that target the negative cell cycle regulators such as p105Rb and p53 are the most important viral oncoproteins during cancer development [18]. Persistent infection with high-risk HPV types is one of the risk factors of cervical cancer [19]. In this study, the overall performance of the HPV DNA testing on MB samples was comparable to HPV PCR on Pap smear samples and histological diagnosis. The sensitivity and PPV of HPV detection using MB compared to a Pap smear were found to be 66.7% in this study. In the study performed by Wong et al. in 2010, the sensitivity, specificity, PPV and NPV of HPV detection using MB were found to be 82.8%, 93.1%, 90.0% and 87.9%, respectively. A total of 558 women were recruited in that study [5,7]. We believe the difference in sensitivity and PPV between the two studies was the result of the small sample size in this study. Poor sample quality during self-collection of MB might also affect the performance of the HPV DNA detection.

In a patient (ST06) who was diagnosed with CIN1/2 and HPV infection, high-risk HPV DNA was also detected in a Pap smear by PCR. However, HPV DNA was not detected in the MB sample. We believe that this discordance between the two HPV DNA test results comes from improper MB collection during sampling. As the patient was instructed to collect her MB sample on the used napkins with a collection foam swab, inaccurate sample collection (e.g., insufficient amount of sample collected, incorrect sample collected, sample contamination, etc.) is possible [20]. In the MB sample ST12, HPV DNA was not detected in the Pap smear by PCR. However, DNA of high-risk HPV type 16 was detected in the MB sample. As for the histological diagnosis, this patient was diagnosed with CIN3 and HPV infection. This shows that the HPV PCR testing on MB samples corelates well with the histological findings. To continue the investigation, further evaluation on the MB collection method (e.g., using a collection swab, napkin or menstrual cup for MB collection) may be required in future to explore and standardize the optimal sample collection method. Clear and detailed instructions should be provided to the patients to collect a high-quality and sufficient amount of the MB sample before sending it to the laboratory for analysis. As for the laboratory testing, quality control (similar to the cellularity control in PCR-based HPV test) should be implemented to ensure that the quality of the specimen is good for analysis.

Regarding the mNGS analysis of MB samples using PacBio 16S rDNA HiFi long-read sequencing, a shift in vaginal flora and a significant depletion in *Lactobacillus* species in the vaginal microbiota communities were observed in all participating patients through mNGS analysis of the MB samples. An increase in microbiota diversity was also observed. Two bioinformatic pipelines (QIIME2 and DADA2) were used in the analysis of amplicon sequence data in this study. The most frequently detected bacteria in the MB samples from HPV-infected women in this study were *Lactobacillaceae*, *Prevotella*, *Gardnerella*, *Peptoniphilus*, *Anaerococcus* and *Streptococcus.* The composition of vaginal microbiota is dynamic and it serves an important role in determining vagina health because it is the first line of defence against infections. A healthy vaginal ecosystem is usually dominated by *Lactobacillus*, with a low diversity of anaerobic bacteria, and a balanced vaginal immune system [21]. Normally, the *Lactobacillus* species, including *Lactobacillus crispatus*, *Lactobacillus gasseri*, *Lactobacillus iners* and *Lactobacillus jensenii,* predominate the vaginal flora. These *Lactobacillus* species protect the vaginal ecosystem and prevent the invasion of pathogens by producing lactic acid that creates a low pH environment [21,22,23,24]. Multiple studies have also shown the association between a disruption in the microbial community in the vagina and increased vaginal infections such as BV, vulvovaginal candidiasis (VVC), STDs, HPV infections, human immunodeficiency virus (HIV) susceptibility and genital herpes infections [25,26,27]. Emerging data indicates that an increased diversity of the vaginal microbiota and a reduced abundance of *Lactobacillus* species due to dysbiosis could contribute to HPV acquisition. Vaginal dysbiosis induces hallmarks of cancer including epithelial barrier disruption, abnormal cellular proliferation, genome instability, angiogenesis, chronic inflammation and metabolic dysregulation, hence the development of cervical cancer if HPV infection is persistent [28]. In this study, all MB samples belong to CST IV B, which is characterized by a low abundance of *Lactobacillus* species and a high diversity and predominance of anaerobic bacteria such as *Gardnerella vaginalis*, *Atopobium vaginae*, *Prevotella* species and other anaerobic bacterial species according to the CST classification [14]. Vaginal microbiome CST is characterized by the type and amount of *Lactobacillus* present. Vaginal CST classification was first proposed by Ravel et al. [14] and supplemented by Gajer et al. [29] afterwards. There are five major types of CST. CST I is considered the healthiest type. It is dominated by *Lactobacillus crispatus*. Women with vaginal CST I are less susceptible to vaginal infections (e.g., BV and STDs) and have the lowest risk of other health complications. CST II is also a healthy type. It is dominated by *Lactobacillus gasseri*. CST III is dominated by *Lactobacillus iners*, a versatile species of *Lactobacillus*. Women with vaginal CST III may have symptoms such as itching and abnormal discharge. CST IV is characterized by a low abundance of lactobacilli and a high diversity of other bacterial species. CST IV is associated with vaginal dysbiosis and a less stable vaginal environment. Women with vaginal CST IV are more prone to recurrent infections and have increased risk of other adverse health outcomes such as pregnancy complications, STD acquisition and PID. CST V is a healthy type, which is dominated by *Lactobacillus jensenii*. Our findings on the mNGS analysis of MB samples matched the clinical conditions of the patients who participated. It also showed that MB is a proper diagnostic specimen for studying the vaginal microbiota using mNGS.

In the MB samples ST06 and ST13, a significant depletion in *Lactobacillus* species was observed and the proportion of *Gardnerella* in the vaginal microbiota communities was found to be 46% and 37%, suggestive of BV. In MB sample ST04, the proportion of other BV-related bacteria such *Sneathia* (19%), *Prevotella* (18%) and *Gardnerella* (7%) was also found to be high. BV is the primary cause of abnormal vaginal discharge or vaginitis resulting from imbalanced vaginal microbiota communities. Lee et al. showed that *Sneathia* species and *Prevotella* species are potential predictors of HPV infection. These bacterial species inhabit the mucous membrane and invade epithelial cells, causing different human infections [30]. Most women suffering from BV are asymptomatic. The diagnosis of BV usually relies on the results of laboratory tests (e.g., isolation of BV-related bacteria by bacterial culture, determining the Nugent score from vaginal Gram stain, the presence of clue cells, etc.) and clinical criteria. The use of lactobacilli and potentially its derivatives (e.g., *Lactobacillus* surface-active molecules) could prevent BV-related vaginal infections via restoration of indigenous microbiota and their anti-biofilm capability [21,28].

In addition to BV-related bacteria, some potential GTI causative pathogens were detected. For example, *Mycoplasmataceae* was detected in ST06, *Enterobacterales* was detected in ST08 and *Streptococcus* was detected in ST10. However, due to the inherent technical limitations of the low taxonomical resolution by 16S rDNA sequencing, accurate species identification was not obtained. Accurate species identification is critical to the clinical management of a patient. For example, in *Mycoplasmataceae*, *Mycoplasma genitalium* is a sexually transmitted pathogen causing cervicitis and PID [31], which may require immediate treatment, while *Mycoplasma hominis* can be part of the normal commensal in the genital tract [32]. As for *Streptococcus* species, *Streptococcus agalactiae* is a critical pathogen that can cause adverse pregnancy, maternal infections, premature delivery, stillbirth and other adverse maternal and infant outcomes [33]. However, the virdans group *Streptococci* is part of the normal commensal and of less clinical significance in the genital tract [34] compared to *Streptococcus agalactiae*. *Enterobacterales* such as *Escherichia coli* and *Klebsiella pneumoniae* are common causative agents of neonatal infections [35,36,37]. Apart from the low taxonomical resolution by 16S rDNA sequencing, sequencing-based methods cannot distinguish living bacteria from dead bacteria [38]. Furthermore, 16S rDNA sequencing cannot provide any information about the antibiotics susceptibility of the pathogens detected. A culture-based method has to be performed. Although 16S rDNA mNGS cannot replace the conventional bacterial culture method at this stage, it is still a promising adjunct diagnostic technique that can be used together with bacterial culture for timely diagnosis and treatment initiation.

To sum up, MB testing is suitable for all women who menstruate as a routine screening method, especially in (1) low-resource countries where the necessary infrastructures are not available; (2) for symptomatic women who are reluctant to consult physicians because of embarrassment or pain; and (3) for women who need more frequent follow-up for treatment success or recovery. There are several inherent limitations in this study. First, the low taxonomical resolution in 16S rDNA sequencing has limited the clinical applications of this technology to accurately identify some clinically important bacteria down to species level (e.g., *Enterobacteriaceae*, *Streptococcus*, *Mycoplasma*, etc.). Additional sequencing or the use of whole-genome sequencing (WGS) might be required for better species resolution. Second, in spite of the small sample size in this study, the results from this study have opened up new possibilities of the use of MB as a diagnostic specimen to maintain women’s health and the use of mNGS as a novel diagnostic tool for a more comprehensive analysis of the vaginal microbiome. Third, the quality of the MB samples collected in this study depended on the skill of the patients during self-collection. Unsatisfactory MB samples collected may lead to inaccurate testing results. Fourth, healthy women were not recruited in this study. However, analysis of the MB samples from healthy women has been reported in our previous studies [6,7].

With the advent of sequencing technologies and the emergence of third-generation NGS platforms such as Oxford Nanopore Technology, the cost of NGS and the sequencing time have been reduced significantly. These advancements allow timely and accurate identification of the causative microbial pathogens, and hence, the proper treatment of infections [39]. In terms of future development, WGS can be used to study the vaginal microbiota communities in MB samples for ideal species identification of clinically important pathogens causing GTIs. NGS can also be applied to HPV genotyping instead of PCR-based methods on MB samples. The applications of NGS-based methods in the detection of STDs and HPV have been widely explored [40,41,42]. However, no investigations on the use of NGS in HPV detection and genotyping have been performed on MB. Moreover, further validation on the diagnosis of STD targets such as *Chlamydia trachomatis*, *Neisseria gonorrhoeae* and *Trichomonas vaginalis* using MB as a potential diagnostic specimen can also be performed to extend the horizon of the use of MB testing. Another promising application of MB testing would be cervical cancer screening and monitoring. Wong et al. showed that MB can be used to detect *TAP1* gene polymorphisms (I333V and D637G). HPV-infected patients with single-nucleotide polymorphisms (SNPs) detected in one or two alleles in the *TAP1* gene have a reduced risk of high-grade cervical intraepithelial neoplasia (HGCIN), and hence require less frequent follow-ups [6]. Regarding the acceptability of using MB as diagnostic samples, Wong et al. evaluated 5000 women’s acceptability on using MB as diagnostic samples. In that study, 87% of the participants supported the use of MB samples for HPV detection compared to the traditional Pap test [6]. Another study performed by Budukh et al. also supports the use of MB as a cervical cancer screening specimen. This practice eliminates not only the discomfort during invasive sample collection but also allows more flexibility for women to manage their daily activities [43]. Urine is another alternative specimen for the detection of HPV DNA non-invasively. First-void urine is often used because it contains more mucus, cell debris and secretions of the uterus, cervix and vagina. These substances usually accumulate between the labia minora and around the urethra opening and are flushed away during urination. However, the instability of HPV DNA in urine is still a concern when urine is used for HPV detection. The amount of human DNA in urine is also variable between samples or individuals and the optimal volume of the urine to be collected has not been established yet [44,45,46]. The performance of HPV DNA detection in urine is better in women with more severe cervical diseases that may cause more exfoliated cervical cells in urine [47]. Overall, the use of MB as a diagnostic specimen of HPV detection and GTI detection may increase women’s participation in cervical cancer screening, as well as GTI screening. The specimen collection procedure is non-invasive when using an MB swab, saves time, and is also convenient.

## 5. Conclusions

In conclusion, our findings have demonstrated that MB is a proper diagnostic specimen of consideration for non-invasive detection of HPV DNA and genotyping using PCR, but it is also a proper diagnostic specimen for the diagnosis of GTIs using mNGS. MB testing is suitable for all women who menstruate and this study has opened up the possibility of the use of MB as a diagnostic specimen to maintain women’s health.

## Figures and Tables

**Table 1:** Summary statistics of PacBio HiFi sequencing data.

Sample ID	Number of Reads	Number of Bases (bp)	N50 Read Length (bp)	Median Read Quality (Phred)
ST04	551,743	823,059,442	1491	42.1
ST06	78,529	117,584,092	1489	43
ST08	370,588	557,280,881	1503	39.9
ST10	357,940	541,838,371	1512	39.1
ST12	295,312	446,999,092	1523	41.8
ST13	47,963	72,772,473	1532	43.9

**Table 2:** The demographic, clinical information of the patients and menstrual blood testing results.

Sample ID	Age	Cytological Diagnosis	Histological Diagnosis	HPV PCR Results in Pap Smear	HPV DNA Detected in MB	Bacterial DNA Detected in MB by mNGS
ST04	34	ASC-US	CIN1	High-risk HPV DNA detected	HPV 16 and 18 DNA detected	Sneathia (19%); Prevotella (18%); Gardnerella (7%); Fastidiosipila (6%); Megasphaera (6%); Parvimonas (5%); Atopobium (4%); Mageeibacillus (2%); Peptoniphilus (2%); Porphyromonas (2%); Dialister (2%); Gemella (2%); Peptostreptococcus (1%); Actinomycetaceae (0.7%)
ST06	45	ASC-US	CIN1/2 HPV infection	High-risk HPV DNA detected	HPV DNA not detected	Gardnerella (46%); Anaerococcus (16%); Prevotella (7%); Peptoniphilus (4%); Dialister (3%); Peptostreptococcus (2%); Atopobium (2%); Streptococcus (1%); Mycoplasmataceae (0.7%)
ST08	43	ASC-H	CIN2	Not done	HPV DNA not detected	Gammaproteobacteria (36%); Enterobacterales (11%); Corynebacterum (6%); Fastidiosipilla (5%); Anaerococcus (0.8%); Facklamia (1%)
ST10	33	LSIL	HPV infection	Not done	High-risk HPV DNA detected (not 16 & 18)	Anaerococcus (6%); Streptococcus (5%); Lactobacillaceae (3%); Prevotellaceae (3%); Corynebacterum (2%); Peptoniphilus (1%); Prevotella (1%)
ST12	39	LSIL ASC-H	CIN3 HPV infection	High-risk HPV DNA not detected	HPV 16 DNA detected	Veillonella (33%); Peptoniphilus (6%); Streptococcus (5%); Lactobacillaceae (2%); Anaerococcus (2%); Prevotella (2%); Corynebacterum (2%); Ureaplasma (1%); Enterobacterales (1%)
ST13	45	LSIL	CIN1 HPV infection	High-risk HPV DNA detected	High-risk HPV DNA detected (not 16 and 18)	Gardnerella (37%); Lactobacillaceae (11%); Streptococcus (2%); Ureaplasma (2%);

ASC-US: atypical squamous cells of undetermined significance; ASC-H: atypical squamous cells, cannot exclude a high-grade squamous intraepithelial lesion; LSIL: low-grade squamous intraepithelial lesion; CIN: cervical intraepithelial neoplasia; Pap smear: papanicolaou smear; HPV: human papillomavirus; MB: menstrual blood; PCR: polymerase chain reaction; mNGS: metagenomic next-generation sequencing.

**Table 3:** The sensitivity and positive predictive value (PPV) of HPV DNA PCR using menstrual blood (MB).

		HPV DNA Detected in MB	HPV DNA Not Detected in MB	Sensitivity	PPV
HPV PCR using Pap smear	HPV DNA detected	2 ^a^	1 ^b^	66.7%	66.7%
	HPV DNA not detected	1 ^c^	N/A		

Note: Sensitivity = [a/(a + c)] × 100%; PPV = [a/(a + b)] × 100%.

## Data Availability

The data presented in this study are available on reasonable request from the corresponding author due to patients’ privacy.
